# Nitrogen addition, not initial phylogenetic diversity, increases litter decomposition by fungal communities

**DOI:** 10.3389/fmicb.2015.00109

**Published:** 2015-02-18

**Authors:** Anthony S. Amend, Kristin L. Matulich, Jennifer B. H. Martiny

**Affiliations:** ^1^Department of Botany, University of Hawaii at ManoaHonolulu, HI, USA; ^2^Department of Ecology and Evolutionary Biology, University of California at IrvineIrvine, CA USA

**Keywords:** climate change, phylogenetic diversity, ecosystem function, fungi, leaf litter decomposition, microcosm, nitrogen fertilization

## Abstract

Fungi play a critical role in the degradation of organic matter. Because different combinations of fungi result in different rates of decomposition, determining how climate change will affect microbial composition and function is fundamental to predicting future environments. Fungal response to global change is patterned by genetic relatedness, resulting in communities with comparatively low phylogenetic diversity (PD). This may have important implications for the functional capacity of disturbed communities if lineages sensitive to disturbance also contain unique traits important for litter decomposition. Here we tested the relationship between PD and decomposition rates. Leaf litter fungi were isolated from the field and deployed in microcosms as mock communities along a gradient of initial PD, while species richness was held constant. Replicate communities were subject to nitrogen fertilization comparable to anthropogenic deposition levels. Carbon mineralization rates were measured over the course of 66 days. We found that nitrogen fertilization increased cumulative respiration by 24.8%, and that differences in respiration between fertilized and ambient communities diminished over the course of the experiment. Initial PD failed to predict respiration rates or their change in response to nitrogen fertilization, and there was no correlation between community similarity and respiration rates. Last, we detected no phylogenetic signal in the contributions of individual isolates to respiration rates. Our results suggest that the degree to which PD predicts ecosystem function will depend on environmental context.

## INTRODUCTION

The ubiquity and abundance of terrestrial fungi is indicative of their pivotal role in providing ecosystem services. It has been estimated that 1 g of soil contains as much as 200 m of fungal hyphae (Leake et al., 2004). In particular, fungi are key players in the degradation of dead plant material (litter), and are capable of breaking down complex carbon sources such as lignin, hemicellulose, and chitin (Lindahl et al., 2007; Allison et al., 2009). Soils contain roughly twice the carbon of either the atmospheric or vegetation pools (Batjes, 1996), and nutrients in the litter layer are presumably the most labile and rapidly cycled. Therefore, changes in litter decomposition rates are likely to have the most immediate impacts on carbon cycling (Cornelissen et al., 2007).

Recent empirical evidence suggests that differences in microbial community composition correlate with differences in community functioning. A handful of studies have examined this relationship using reciprocal transplants (Balser and Firestone, 2005; Strickland et al., 2009; Cleveland et al., 2013) or community filtering methods (Griffiths et al., 2000; Austin et al., 2006) and have found significant differences among community responses and process rates (but see Wertz et al., 2007). A few other studies have demonstrated a positive relationship between microbial species richness and community functioning by creating *de novo* assemblages of isolated microorganisms (Naeem et al., 2000; Bell et al., 2005). Presumably, the basis of this relationship is the positive correlation between the number of species and the variety of different, perhaps complementary, traits that contribute to a functional process.

In theory, a community spanning greater evolutionary history – i.e., encompassing higher phylogenetic diversity (PD) – ought to contain a greater number of non-redundant traits. Indeed, recent work suggests that phylogenetic relatedness among plant species is correlated with their trait similarity, leading to a positive relationship between a plant community’s PD and its productivity (Cadotte et al., 2008; Flynn et al., 2011). Many microbial traits are phylogenetically patterned as well (McGuire et al., 2010; Treseder et al., 2011; Lennon et al., 2012; Martiny et al., 2013). In fact, a comparative genomic analysis demonstrated some phylogenetic conservatism for extracellular enzymes (Zimmerman et al., 2013) and glycoside hydrolases (Berlemont and Martiny, 2013), examples of traits that could directly influence litter decomposition rates. Similarly, the ability of leaf-decomposer fungi to metabolize various organic nitrogen compounds seems to be genetically correlated (McGuire et al., 2010). These results suggest that not only are leaf litter fungi functionally distinct, but that PD might be a better predictor of decomposition rate than taxonomic diversity in and of itself.

The relationship between microbial PD and ecosystem functioning is particularly important in light of global change. Many studies demonstrate that fungal communities are sensitive to global change (Avis et al., 2008; Andrew and Lilleskov, 2009; Dang et al., 2009; Edwards et al., 2011; Edwards and Zak, 2011; Kerekes et al., 2013), and that once disturbed, microbial communities do not often rapidly recover to their original state (Allison and Martiny, 2008). Moreover, microbial response tends to be patterned by phylogeny, such that perturbed communities consist of more closely related species than would be expected by chance (Placella et al., 2012; Evans and Wallenstein, 2014). Specifically, drought and thermal tolerance appears to be phylogenetically patterned at the phylum level (Treseder et al., 2014).

Overall, fungal traits, including those involved in decomposition and in the response to changing environments, appear to be phylogenetically conserved. Thus, we first test the hypothesis that PD of the fungal species pool is positively correlated with community functioning, measured here as litter respiration. We combine 42 fungal species isolated from a natural litter ecosystem, spanning approximately 600 million years of evolutionary history, into a series of communities along a PD gradient. We hold initial species richness constant to control for portfolio effects as richer communities are more likely to contain better competitors (Tilman, 1999). We further hypothesize that PD, by increasing the breadth of a community’s traits, will also buffer a community’s sensitivity to environmental change. To test this hypothesis, we fertilized a subset of the microcosm communities with nitrogen, one aspect of ongoing environmental change in the grassland ecosystem from which the fungi were sampled (Fenn et al., 2010). We predicted that differences in decomposition rates between fertilized and ambient microcosms would be inversely proportional to the PD of its community.

## MATERIALS AND METHODS

### SAMPLING AND FUNGAL ISOLATION

Leaf litter was collected from a grassland savannah located near Irvine, CA, USA (33.74 N, 117.70 W), described in detail elsewhere (Allison et al., 2013). The site is dominated by invasive grasses and forbs. The same leaf litter was divided into two portions for fungal isolations and the microcosm experiment. For isolations, leaf litter was homogenized in a sterile coffee grinder, and filtered through sequential 2 mm, 212 μm, and 106 μm prefilters. The 106–212 μm size fraction was transferred to a sterile 100 μm nylon vacuum filter, washed twice in 200 mL sterile H_2_0, and transferred to 30 ml 0.6 carboxymethylcellulose solution (an emulsifier). The filtrate was sequentially diluted until 50% of 10 μl aliquots yielded either 0 or 1 fungal colony after 1 week of incubation. Ten microliter aliquots of filtrate were added to 800, 1 ml titer tubes containing 500 μl of solid MEA, water, MNM, or Thorn’s medium (Thorn et al., 1996) amended with Kanamycin and Ampicillin (200 tubes per medium). Tubes were incubated at room temperature until growth was detected.

### FUNGAL IDENTIFICATION, SEQUENCING, AND COMPARISON WITH CULTIVATION INDEPENDENT FIELD DATA

Isolates were sorted into visually distinct morphotypes, and a representative of each was PCR amplified using the primers ITS1f-TW13 (White et al., 1990; Gardes and Bruns, 1993), which spans ∼1,400 bp, including both ITS spacers and the D1 and D2 regions of the gene encoding for the large ribosomal RNA subunit. Amplicons were sequenced in two directions using the sequencing services of Beckman Coulter, using the same PCR primers. For taxon circumscription and identification, the ITS spacers were excised from adjacent 18s, 5.8s, and 28s gene regions using an algorithm based on Hidden Markov Models (ITSx; Bengtsson-Palme et al., 2013) and concatenated and clustered into groups containing 97% sequence identity or greater using Sequencer’s (version 4.7; Gene Codes) “contig” function. A single isolate from each contig was selected for subsequent analysis. Sequences are deposited in Genbank under accession numbers KF733341–KF733375.

Taxa were compared to a distribution of fungal communities enumerated using 454 sequencing technology from the field site over a 2-years sampling period (as described in Matulich and Martiny, 2014). Portions of the 28s encoding gene were matched to environmental DNA sequences at 97% sequence identity using the nearest neighbor clustering algorithm of the UCLUST package (Edgar, 2010). Taxonomic assignments of the environmental sequences were determined using the RDP fungal LSU classifier (Liu et al., 2012).

### PHYLOGENETIC TREE

**FIGURE 1:**
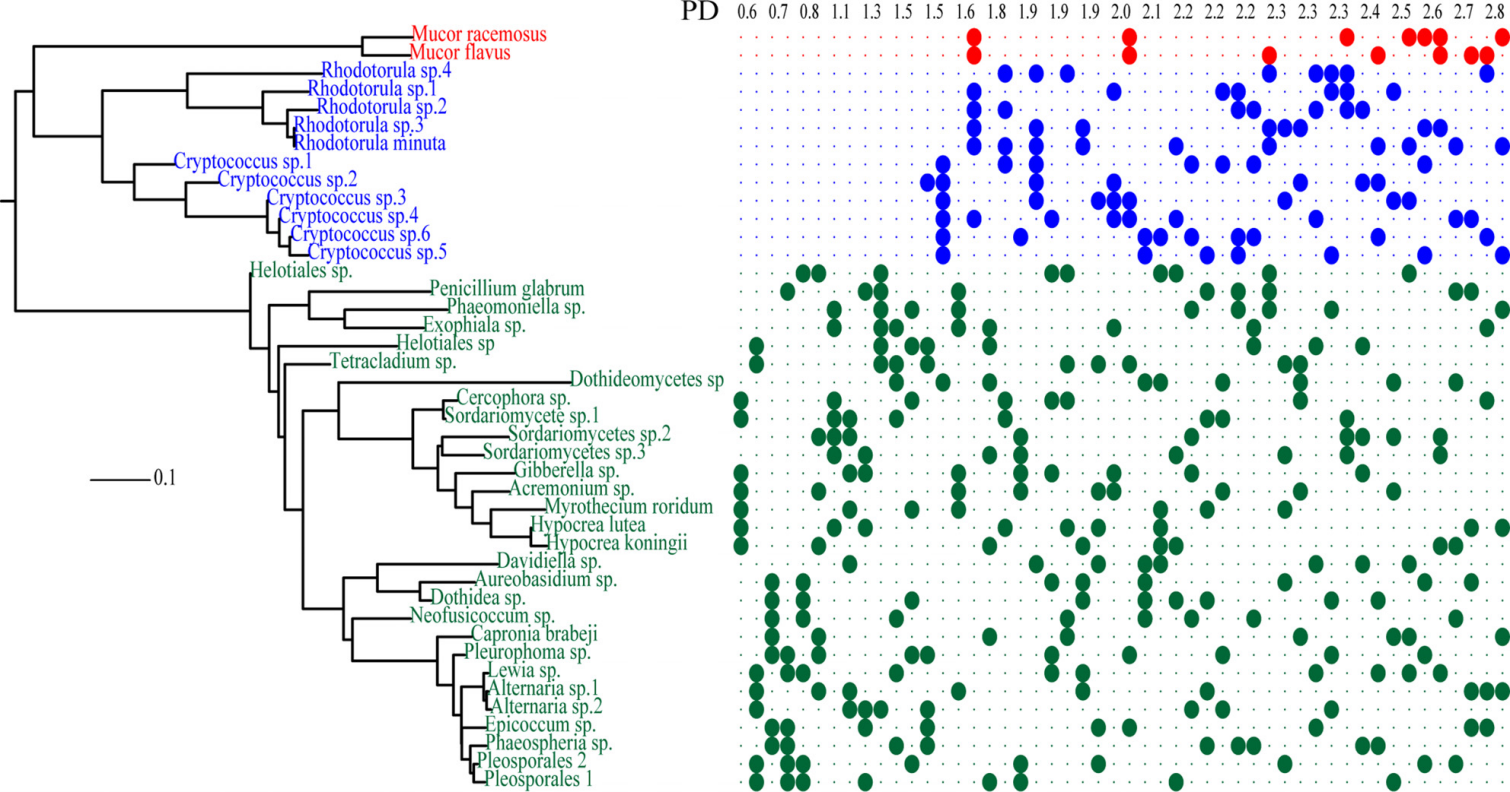
**Maximum likelihood tree and community composition of microcosms.** Each column of the grid indicates the composition of one of 50 mock communities consisting of seven species in total. Species are colored by taxonomy as follows: Ascomycetes (green), Basidiomycetes (blue), and Mucoromycetes (red). Communities are ordered by increasing phylogenetic diversity (PD) as indicated by the scale at top.

The 28s portions of the sequences (and outgroups *Spizellomyces punctatus* and *Rozella allomycis*) were aligned using MAFFT’s L-INS-i algorithm (Katoh et al., 2009), and a maximum likelihood tree was calculated in RaXML (Stamatakis et al., 2008) on the CIPRES server using default settings (**Figure 1**).

### COMMUNITY ASSEMBLY AND MICROCOSM CONSTRUCTION

Fifty distinct species pools were assembled along a PD gradient such that each microcosm contained seven taxa, each taxon was present in 7–10 communities, and no community shared more than three taxa. Isolates were selected to enable phylogenetically nested species pools containing both closely related congeners as well as distinct phyla (**Figure 1**).

Leaf litter for microcosms was homogenized in a Wiley mill, and sterilized via gamma irradiation for 48 h. Sterility was verified by plating litter on nutrient media. Selected fungal isolates were transferred to petri dishes containing growth medium with 5 mm cellophane disks on top, over which fungal colonies grew. Ten cellophane disks per isolate were transferred to a tube containing sterile water and one 3 mm silica bead and were briefly vortexed to suspend fungal cells. Microcosms were established in 40 ml sterile vials with gas-tight septum caps filled with 2 g sterile sand, 200 mg leaf litter substrate and 40 μl of fungal slurry for each species (280 μl total). Each community was replicated six times, and half of the replicates received a supplement of 71.4 μg NH_4_NO_3_. We estimate that this amount represents a litter C: fertilizer N ratio of approximately 5,000, equivalent to roughly 0.2 kg/ha. This is an ecologically relevant amount of Nitrogen that is lower than typical field deposition trials (Allison et al., 2009), and represents less than 10% annual deposition in this location (Fenn et al., 2010). Fungus free control microcosms were also run with and without nitrogen addition, replacing sterile H_2_O for fungal slurry volume.

### MEASUREMENT OF CO_2_ PRODUCTION

CO_2_ mineralization rate, our proxy metric for litter decomposition, was measured as the amount accumulated in the microcosm headspace over 24 h. Concentrations were measured after the first and third days and then weekly for a total of 66 days. The day prior to each measurement, microcosms were opened under sterile conditions, equilibrated with ambient air for 5 min, and then sealed. For each measurement, an 8 ml subsample of headspace gas was withdrawn by syringe and injected into an infrared gas analyzer (PP-Systems EGM-4). After measurement, vials were vented by rotating caps ¼ turn until 24 h prior to subsequent measure. A different syringe was used for each community to prevent cross-contamination.

### STATISTICAL ANALYSES

We assessed the interactions between decomposition rates and PD using a repeated measures ANCOVA model with sampling day (factor 14 levels) and nitrogen addition (factor 2 levels) as fixed effects, initial PD as a covariate, and community composition (factor 50 levels) as a random effect. PD was calculated three ways: (1) as a measure of the cumulative phylogenetic branch length (PD) contained amongst all community members, (2) as a measure of the nearest taxon index (NTI), which is the mean phylogenetic distance between all taxa and their closest relatives in a community, and (3) as the net relatedness index (NRI) which is the mean phylogenetic distance between all pairs of taxa within a community. All indices were calculated in the R package “picante” (Kembel et al., 2010), and the ANCOVA model was built using the stats package in the R programming environment (R Core Team, 2013).

To test whether there was a correlation between similarity of initial community composition and rates of respiration we used a Mantel test with 999 randomized permutations to assess significance levels. A pseudo cumulative respiration value was calculated by summing 24 h sample time points multiplied by the number of days preceding the last sample. Pairwise differences in cumulative measured CO_2_ were tested for correlation with shared community membership (Jaccard’s index) and shared phylogenetic branch length (Unifrac). We calculated correlations for nitrogen addition microcosms, ambient microcosms, and both together using the R package “vegan” (Oksanen et al., 2013).

To determine whether individual species were significantly associated with increased or decreased respiration rates (compared to average) we calculated a multiple linear regression model. Each species was considered a factor with two levels (present or absent). A dummy species representing mean CO_2_ production across all communities was added as a reference.

To the determine whether individual species contributed to differences in community response with nitrogen addition, a multiple linear regression was calculated as above, substituting cumulative CO_2_ with the proportional difference between treatments (ambient/nitrogen fertilization). Models were validated by plotting residuals vs. fitted values, and normal quantile–quantile plots. Models were made using the “stats” package in the R programming environment (R Core Team, 2013), and data was formatted using the package “reshape2” (Wickham, 2007).

Coefficients from these linear multiple regression tests were tested for phylogenetic signal using Blomberg’s K statistic (Blomberg et al., 2003) using the “picante” package (Kembel et al., 2010). This test measures whether variance among taxa differs from expectations given a Brownian motion evolutionary model. Values <1 indicate greater variance than expected whereas values >1 indicate phylogenetic signal, with significance determined by comparing the observed variance distribution with 999 randomizations.

## RESULTS

### EFFECTS OF PHYLOGENETIC DIVERSITY AND NITROGEN FERTILIZATION ON DECOMPOSITION RATES

We found that all three measures of PD were highly correlated (PD-NTI *R*^2^ = 0.86; NTI-NRI *R*^2^ = 0.71; NRI-PD *R*^2^ = 0.96) and selection of one vs. another had no impact on the significance of any results. Therefore, only the results of PD are reported here.

Community PD was not correlated with respiration nor interacted with any other component of the experiment. Instead, nitrogen fertilization, and its interaction with time, appeared to drive respiration rates, with the earliest sampling dates of nitrogen-fertilized microcosms showing the highest levels of respired carbon (**Table 1**).

**Table 1:** Results of the ANCOVA Model testing the effects of phylogenetic diversity (PD), nitrogen addition and sampling time on respiration (top), and on the affects of nitrogen addition on community respiration (cumulative difference; below).

Variable	Factor	Df	Sum squares	Mean squares	*F*	*P*-value
Respiration	PD	1	89969	89969	0.029	0.866
	Residuals	48	150170078	3128543		
	Nitrogen	1	5.76E + 07	57603071	99.018	**< 0.001**
	Time	10	1.53E + 09	153271897	263.469	**<0.001**
	PD:nitrogen	1	1.14E + 05	114248	0.196	0.658
	PD:time	10	3.01E+ 06	300580	0.517	0.879
	Nitrogen:time	10	7.71E + 07	7707967	13.25	**<0.001**
	PD:nitrogen:time	10	1.43E + 06	142590	0.245	0.991
	Residuals	1008	5.86E + 08	581745		
Cumulative difference	PD	1	0.37	0.369	0.071	0.791
	Residuals	48	248.77	5.183		
	Time	10	1.00E-27	1.00E-28	1.864	**0.048**
	PD:time	10	1.04E-28	1.04E-29	0.193	0.997
	Residuals	480	2.58E-26	5.37E-29		

*Bolded values indicate statistical significance at 0.05.*

**FIGURE 2:**
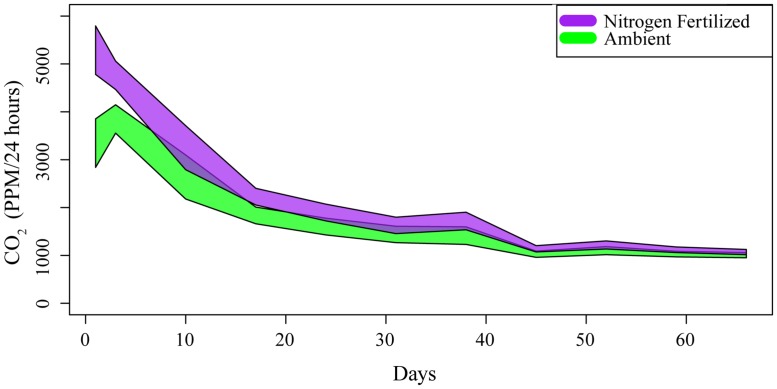
**Time series of mineralized CO_**2**_, a proxy for decomposition.** Each polygon contains two SE of the mean amongst replicates for each community under ambient and nitrogen fertilized conditions. Units are parts per million (PPM) of CO_2_ accumulated in microcosms over 24 h.

Phylogenetic diversity also did not correlate with difference in respiration rate between fertilized and ambient microcosms (ANCOVA; *F*_1_ value: 0.071, *P* = 0.791). Differences did correlate with sampling date, however. Nitrogen addition had the greatest impact during the earliest sampling dates, with the differences in respiration between ambient and fertilized microcosms diminishing over the course of the experiment (*F*_10_ value: 1.864, *P* = < 0.048). In nearly all cases, rates of decomposition peaked between days one and three and steadily declined throughout the duration of the experiment (**Figure 2**).

### COMMUNITY COMPOSITION AND RESPIRATION RATES

Community composition was not correlated with cumulative respiration, whether using either a measure of shared taxa (Mantel_Jaccard_ *r* = -0.017, *P* = 0.17) or a measure of shared phylogenetic branch length (Mantel_UniFrac_ *r* = -0.025, *P* = 0.621). This was the case whether we considered the ambient or nitrogen fertilized treatments together or individually.

### CONTRIBUTION OF INDIVIDUAL TAXA TO COMMUNITY DECOMPOSITION RATES

**FIGURE 3:**
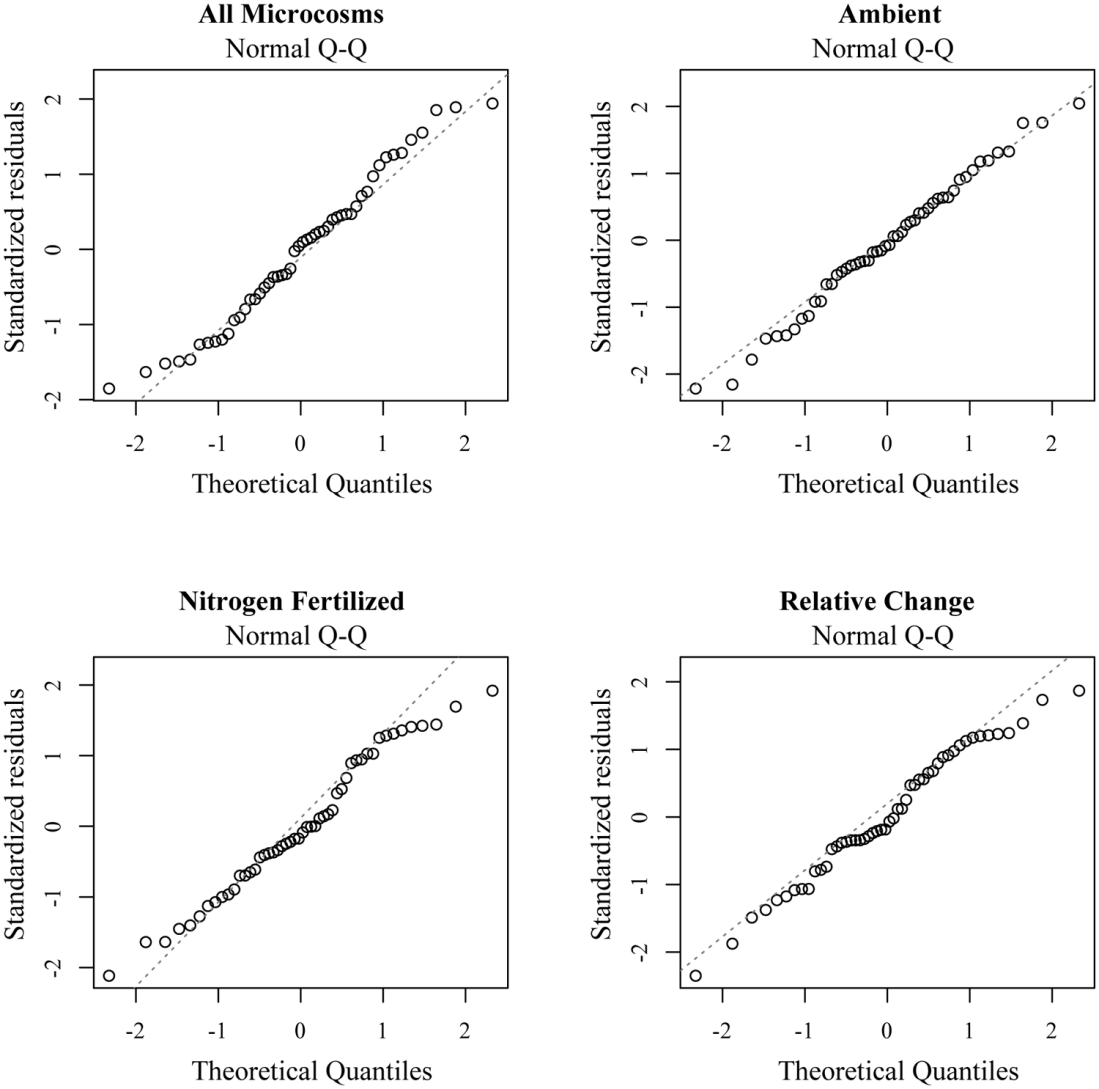
**Quantile–quantile (Q–Q) normality plots indicating normal residuals for all four response variables**.

Individual contributions to community respiration rates were measured using a general linear model in which the coefficient associated with each of the species indicates its contribution to respiration or to the difference between decomposition in the fertilized and control microcosms. The coefficients are approximately normally distributed (**Figure 3**), indicating that each species was effectively equivalent with a few notable exceptions. Non-fertilized microcosms containing *Cryptococcus* sp. 6, *Rhodotorula* sp. 3, and *Hypocrea lutea* showed significantly slower rates of respiration compared to average, whereas none of these species appeared to impact fertilized microcosms (**Table 2**). Conversely, *Cercophora* sp. was correlated with significantly higher rates of respiration in ambient microcosms.

**Table 2:** Results from the multiple regression analyses of species effects on decomposition, from left to right: all microcosms combined, ambient, nitrogen fertilized, and the effect size of fertilization.

Species	Combined *T*-value	Ambient *T*-value	*N* + *T*-value	Difference *T*-value
*Rhodotorula*_sp.1	0.603	0.073	0.778	0.699
*Rhodotorula*_sp.3	-1.136	-2.391*	-0.065	**1.931**
*Rhodotorula_minuta*	-0.019	0.855	-0.558	-1.592
*Rhodotorula*_sp.2	1.08	1.687	0.426	-0.757
*Rhodotorula*_sp.4	-1.109	-1.414	-0.635	0.315
*Cryptococcus*_sp.1	0.908	0.972	0.636	-0.161
*Cryptococcus*_sp.2	0.115	0.482	-0.143	-0.835
*Cryptococcus*_sp.3	0.215	0.166	0.191	0.026
*Cryptococcus*_sp.6	-1.57	-3.552**	0.065	3.169*
*Cryptococcus*_sp.5	0.601	1.136	0.114	-1.398
*Cryptococcus*_sp.4	-1.413	-1.66	-0.898	0.331
*Mucor_racemosus*	-0.39	-0.656	-0.125	0.438
*Mucor_flavus*	1.459	1.814	0.865	-0.995
Helotiales_sp.	1.008	0.835	0.857	0.527
*Penicillium_glabrum*	-0.766	-1.723	0.026	0.687
*Phaeomoniella_*sp.	-0.088	0.418	-0.381	-0.04
*Exophiala_*sp.	-0.33	-0.582	-0.089	-0.302
Helotiales_sp	-0.898	-1.481	-0.305	0.325
*Tetracladium_*sp.	0.421	0.158	0.477	0.236
Dothideomycetes_sp	-0.884	-0.138	-1.122	-1.112
*Cercophora_*sp.	**1.875**	2.572*	0.961	-1.434
Sordariomycete_sp.1	-0.631	-1.166	-0.136	1.016
Sordariomycetes_sp.2	-0.95	-0.597	-0.926	-0.914
Sordariomycetes_sp.3	1.75	1.465	1.48	-0.38
*Gibberella_*sp.	0.207	-0.639	0.681	1.254
*Hypocrea_lutea*	-1.566	-2.512*	-0.577	**1.27**
*Hypocrea_koningii*	0.214	0.069	0.25	0.509
*Myrothecium_roridum*	-0.115	0.26	-0.318	-0.689
*Acremonium_*sp.	-0.002	0.724	-0.453	-1.262
*Davidiella_*sp.	1.248	0.649	1.301	0.468
Aureobasidium_sp.	-0.062	0.257	-0.244	-0.411
*Dothidea_*sp.	0.444	0.56	0.259	-0.159
*Lewia_*sp.	-0.662	-0.807	-0.402	-0.414
*Alternaria_*sp.1	0.988	0.375	1.117	0.876
*Alternaria_*sp.2	-0.088	0.005	-0.123	-0.229
*Phaeosphaeria_*sp.	-0.43	-0.619	-0.202	0.327
Pleosporales_2	0.746	0.937	0.436	-0.084
Pleosporales_1	-0.009	0.646	-0.414	-0.634
*Epicoccum_*sp.	-0.007	0.506	-0.325	-0.778
*Pleurophoma_*sp.	-0.262	-1.211	0.395	1.173
*Capronia_brabeji*	-0.736	-1.609	-0.004	0.725
*Neofusicoccum_*sp.	**1.998**	1.846	1.581	-0.011
**Blomberg’s *K***	0.00061	0.00003	0.00025	0.00001

Blomberg’s *K* statistic of phylogenetic signal and its *P*-value are reported below. Significance values are reported as: *P < 0.05, **P < 0.01, **Bold** no asterisk = <0.10.

Blomberg’s *K* test for phylogenetic signal between correlation coefficients and phylogenetic relatedness were near zero for all groups of microcosm (ambient, fertilized, combined) and none were significantly different from random expectations (**Table 2**). In fact, species within the genus *Cryptococcus* contained both the lowest and fourth highest coefficient scores.

### ABUNDANCE OF ISOLATES IN THE FIELD SURVEY

Thirteen of 42 isolates were detected in the field survey, comprising 0.91% of the total sequence abundance (**Table 3**). The single most abundant taxon isolated was *Cryptococcus* sp. 3, accounting for 0.669% of the total sequence abundance. Family level taxonomy was determined for 30 isolates, all of which were detected in the field survey. Cumulatively these families contained >88% of all sequence abundance, and were dominated by the Davidiellaceae and the Pleosporaceae (comprising 39.0 and 37.6% of the relative abundance, respectively).

**Table 3:** Relative abundance of isolates and isolate families in a field survey of leaf litter fungi.

Isolate name	Family (unless otherwise noted)	Phylum	Taxon relative abundance in survey	Family relative abundance in survey
*Tetracladium* sp.	Ascomycota_incertae_sedis (phylum)	Ascomycetes	–	N.D.
*Neofusicoccum* sp.	Botryosphaeriaceae	Ascomycetes	–	0.191
*Davidiella* sp.	Davidiellaceae	Ascomycetes	–	39.027
*Dothidea* sp.	Dothideaceae	Ascomycetes	–	0.634
Dothideomycetes *sp.*	Dothideomycetes (class)	Ascomycetes	–	–
*Aureobasidium* sp.	Dothioraceae	Ascomycetes	–	0.863
Helotiales sp.	Helotiales (rank)	Ascomycetes	–	–
Helotiales sp.	Helotiales (rank)	Ascomycetes	–	–
*Myrothecium roridum*	Helotiales_incertae_sedis (rank)	Ascomycetes	–	N.D.
*Capronia brabeji*	Herpotrichiellaceae	Ascomycetes	–	0.026
*Exophiala* sp.	Herpotrichiellaceae	Ascomycetes	0.008	0.026
*Phaeomoniella* sp.	Herpotrichiellaceae	Ascomycetes	0.000	0.026
*Hypocrea koningii*	Hypocreaceae	Ascomycetes	–	0.000
Hypocreales sp.	Hypocreaceae	Ascomycetes	0.000	0.000
*Acremonium* sp.	Hypocreales_incertae_sedis (rank)	Ascomycetes	–	N.D.
*Cercophora* sp.	Lasiosphaeriaceae	Ascomycetes	–	0.451
*Gibberella* sp.	Nectriaceae	Ascomycetes	0.029	0.102
*Phaeosphaeria* sp.	Phaeosphaeriaceae	Ascomycetes	–	6.770
*Alternaria* sp. 1	Pleosporaceae	Ascomycetes	–	37.611
*Alternaria* sp. 2	Pleosporaceae	Ascomycetes	–	37.611
*Epicoccum* sp.	Pleosporaceae	Ascomycetes	–	37.611
*Lewia* sp.	Pleosporaceae	Ascomycetes	–	37.611
Pleosporales sp. 1	Pleosporales (rank)	Ascomycetes	–	–
Pleosporales sp. 2	Pleosporales (rank)	Ascomycetes	–	–
*Pleurophoma* sp.	Pleosporales_incertae_sedis (rank)	Ascomycetes	0.003	N.D.
Sordariomycetes sp. 1	Sordariomycetes (class)	Ascomycetes	0.190	–
Sordariomycetes sp. 2	Sordariomycetes (class)	Ascomycetes	Singleton	–
Sordariomycetes sp. 3	Sordariomycetes (class)	Ascomycetes	–	–
*Penicillium glabrum*	Trichocomaceae	Ascomycetes	Singleton	0.027
*Rhodotorula minuta*	Sporidiales incertae sedis (rank)	Basidiomycetes	*–*	N.D.
*Rhodotorula* sp.1	Erythrobasidiaceae	Basidiomycetes	–	N.D.
*Rhodotorula* sp. 2	Erythrobasidiaceae	Basidiomycetes	<0.001	N.D.
*Rhodotorula* sp. 3	Erythrobasidiaceae	Basidiomycetes	–	N.D.
*Rhodotorula* sp. 4	Erythrobasidiaceae	Basidiomycetes	0.017	N.D.
*Cryptococcus* sp. 1	Tremellaceae	Basidiomycetes	–	3.124
*Cryptococcus* sp. 2	Tremellaceae	Basidiomycetes	–	3.124
*Cryptococcus* sp. 3	Tremellaceae	Basidiomycetes	0.669	3.124
*Cryptococcus* sp. 4	Tremellaceae	Basidiomycetes	0.000	3.124
*Cryptococcus* sp. 5	Tremellaceae	Basidiomycetes	–	3.124
*Cryptococcus* sp. 6	Tremellaceae	Basidiomycetes	–	3.124
*Mucor flavus*	Mucoraceae	Zygomycota	–	0.000
*Mucor racemosus*	Mucoraceae	Zygomycota	Singleton	0.000

*Singletons are found once in the dataset. N.D. indicates that family level taxonomy is uncertain for the genus.*

## DISCUSSION

The species richness–function relationship presumes a linkage between a species and trait diversity. Under this model taxa are functionally variable and the sum of individual species contributes to combined community functioning. We hypothesized that, due to the tendency of close fungal relatives to contain a more similar suite of traits, PD of litter fungi would be a better predictor of functioning (respiration rate) than taxonomic diversity alone, as has been shown recently amongst communities of marine bacteria (Gravel et al., 2012; Venail and Vives, 2013).

Contrary to our prediction, we found no evidence, by any measure, for a relationship between PD and respiration rates. Initial PD of microcosm taxa pools did not correlate with respiration at any time point in the experiment. Furthermore, although nitrogen fertilization increased respiration rates, this response was independent of phylogenetic community composition: there was no correlation between PD and community resilience. Last, we did not find a phylogenetic signal amongst isolate contributions to community respiration. While previous studies have found significant differences among decomposition rates of fungal isolates (Allison et al., 2009), and some degree of phylogenetic patterning among their substrate utilization (McGuire et al., 2010), we found very few species in our study that correlated with increased or decreased rates of CO_2_ production.

We can think of at least three reasons for the discrepancy between these past results and the present study. First, the scale of PD considered might matter for the diversity-function relationship. Many of the microbial traits examined are phylogenetically conserved, but at a fine genetic scale (e.g., Martiny et al., 2013). Thus, the scale of PD considered here, spanning three phyla, may not be informative. Constraining communities to phylogenetically narrower membership more consistent with detected levels of trait conservatism may be more conducive to detecting a PD–function relationship.

Second, traits of single isolates may be more likely to show a phylogenetic signal than when they are measured within a community context. Together with other taxa, the isolates do not necessarily perform at their functional potential, but are constrained by interactions with the rest of the community. For fungi, competitive interactions between non-self mycelium, including chemical and physical antagonism, can impact resource allocations and decrease decomposition rates (Boddy, 2000). Similarly, synergistic biotic interactions such as complementary abilities to degrade complex or recalcitrant biomolecules such as lignin among Basidiomycetes (Blanchette, 1991), or specialized enzyme production to decompose cellulose and chitin molecules (Lindahl and Finlay, 2006), may accelerate rates of decomposition.

A third potential reason for a lack of correlation between initial PD and functioning is that the realized PD of the microcosms may have differed from the initial PD. Fungal composition may have changed, perhaps rapidly, due to biotic interactions, nutrient availability, and stochastic processes favoring growth of one species over another (Cleveland et al., 2013; Matulich and Martiny, 2014). A previous study with some of the same fungal isolates did observe changes in community structure over the course of a similar, 60 day microcosm experiment (Matulich and Martiny, 2014). Those community changes were largely driven by changes in relative abundance rather than extinctions, and all measured isolates were able to survive under experimental conditions. In fact a vast, and contradictory, literature predicts both the competitive exclusion of and niche selection for closely related organisms (Maherali and Klironomos, 2007; Mayfield and Levine, 2010; Venail and Vives, 2013; Godoy et al., 2014), making it difficult to predict the outcome of biotic interactions based on relatedness alone.

Although initial PD did not alter respiration rates, nitrogen fertilization significantly increased rates, regardless of phylogenetic relatedness or taxonomic composition of microcosms. Earlier research has demonstrated mixed effects of nitrogen availability on decomposition rates, with impacts varying across substrate, taxonomy and functional guild of the microbes under study (Knorr et al., 2005; Allison, 2012). Thus, it appears that community response to nitrogen, and therefore its correlation with phylogenetic patterning, is not consistent across environments, but is context dependent. In low nitrogen environments, for example, nitrogen fertilization has been shown to decrease plant tissue C:N ratios (Bragazza et al., 2011), increase decomposition of cellulose and mineral forms of N (Talbot and Treseder, 2012), and facilitate transcription of lignocellulolytic enzyme genes (Edwards et al., 2011).

Although there is likely to be a mismatch between microbial diversity in natural systems and that amenable to cultivation on lab media, our efforts increased the likelihood that isolate functional and taxonomic diversity were broadly representative of field conditions. Use of multiple media, isolation of a size fraction >100 μm, and dilution to extinction protocols facilitated cultivation of slow growing and less competitive taxa. Further, because the taxa were isolated from the same substrate used in the microcosms, there is the strong likelihood that these fungi are associated with leaf litter decay processes. Cultivation-independent sequence analysis of this field site uncovered more than 800 fungal taxa, of which our isolates comprised approximately 1% of the sequence abundance: a reasonable representation given the typically long-tailed community rank abundance curve. Further, family level taxonomic identities of our isolates represented >88% of the sequence abundance in our field site, indicating that isolate diversity was representative at higher taxonomic ranks.

The complexity of biotic and environmental interactions scales with community and litter complexity and may be difficult to predict. The high species diversity detected in our field site enable an almost unfathomably tangled network of interactions, undoubtedly unique to this site. For this reason, this study highlights the importance of examining traits, particularly those relating to ecosystem function, within the context of the community in which they’re found, rather than in isolation. In a recent study of petroleum degrading bacteria, for example, a positive PD ecosystem function relationship was found in both two and four isolate microcosms (Venail and Vives, 2013). Amongst the latter, an increase in positive biotic interactions underpinned this relationship. However, in a natural microbial community, particularly one as species rich as that found within leaf litter, the average distance between community members will decrease as a function of species richness. Therefore, complementarity will be balanced, at some point, by competition amongst species whose niche requirements overlap. Determining this “tipping point” may be a fruitful endeavor for future research into the PD ecosystem function relationship.

## AUTHOR CONTRIBUTIONS

ASA and JBHM designed the experiment, ASA and KLM conducted the experiment, ASA analyzed the data and all authors contributed towards writing and editing the manuscript.

## Conflict of Interest Statement

The authors declare that the research was conducted in the absence of any commercial or financial relationships that could be construed as a potential conflict of interest.
